# Field size effects on DNA damage and proliferation in normal human cell populations irradiated with X-ray microbeams

**DOI:** 10.1038/s41598-021-86416-7

**Published:** 2021-03-26

**Authors:** Mitsuaki Ojima, Atsushi Ito, Noriko Usami, Maki Ohara, Keiji Suzuki, Michiaki Kai

**Affiliations:** 1 Department of Environmental Health Science, Oita University of Nursing and Health Sciences, 2944-9 Megusuno, Oita, 840-1201 Japan; 2 School of Engineering, Tokai University, Hiratsuka, Kanagawa 259-1292 Japan; 3 Photon Factory, Institute of Materials Structure Science, KEK, Tsukuba, Ibaraki 305-0801 Japan; 4 Department of Radiation Medical Sciences, Atomic Bomb Disease Institute, Nagasaki University, Nagasaki, 852-8523 Japan

**Keywords:** Cell biology, Biological physics

## Abstract

To clarify the health risks of internal radiation exposure, it is important to investigate the radiological effects of local exposure at cell levels from radioactive materials taken up by organs. Focusing on the response of cell populations post-irradiation, X-ray microbeams are very effective at reproducing the effects of local exposure within an internal exposure in vitro. The present study aims to clarify the effects of local exposure by investigating the response of normal human cell (MRC-5) populations irradiated with X-ray microbeams of different beam sizes to DNA damage. The populations of MRC-5 were locally irradiated with X-ray microbeams of 1 Gy at 0.02–1.89 mm^2^ field sizes, and analyzed whether the number of 53BP1 foci as DSB (DNA double strand break) per cell changed with the field size. We found that even at the same dose, the number of DSB per cell increased depending on the X-irradiated field size on the cell population. This result indicated that DNA damage repair of X-irradiated cells might be enhanced in small size fields surrounded by non-irradiated cells. This study suggests that X-irradiated cells received some signal (a rescue signal) from surrounding non-irradiated cells may be involved in the response of cell populations post-irradiation.

## Introduction

Insoluble radioactive cesium was released into the atmosphere due to the Fukushima Daiichi Nuclear Power Plant accident that occurred after the Great East Japan Earthquake on March 11, 2011^1^. Among the isotopes, Cs-137 has a half-life of 30 years and exists as being adsorbed to soil and dust^2^. When Cs-137 is taken into the body though food and respiration, it deposits unevenly in the lungs, causing internal exposure (local exposure in the lungs)^3^.

Currently, the ICRP (International Commission on Radiological Protection) assumes that the cancer risk of internal exposure is in accord with that of external exposure (uniform exposure) if the average absorbed doses in tissues or organs is the same irrespective of heterogeneous distribution^4^. In terms of radiological protection, this approach is the basis for the definition of the protection quantities which are used for limiting stochastic effects and are based upon the assumption of a linear-non-threshold, dose-response relationship (LNT model)^5^. However, there is no biological explanation that the absorbed dose of individual cells composing an organ differs between internal and external exposure.

In an in vitro study using Cs-bearing particles composed of 92.4% ^137^Cs with 469.2 Bq and 7.6% ^134^Cs with 38.5 Bq, Matsuya et al. calculated by Monte Carlo code of PHITS (particle and heavy ion transport code system) the absorbed dose rates around the Cs-bearing particle^6^. They showed that the absorbed dose rate gradually decreased depending on the distance from the Cs-bearing particle, and there was a low absorbed dose rate of below 0.902 mGy/day at 1.65 mm from the particle. This result suggests that cells close to the radiation source receive a high dose and cells far from the radiation source receive a low dose even within the same organ. Therefore, it is expected that internal exposure will have less of an effect on organs than external exposure.

In 1984, Peel et al. analyzed the incidence of skin disorders in circular areas of pig skin from 1 to 40 mm in diameter that were irradiated with β-rays from ^90^Sr, ^170^Tm, and ^147^Pm, respectively^7^. They showed that the doses required to produce moist desquamation in 50% of the skin fields were 30 Gy for the 22.5-mm source, 45 Gy for the 11-mm source, 70 Gy for the 5-mm source, 125 Gy for the 2-mm source, and 450 Gy for the 1-mm source. This result suggested that, even with the same absorbed dose on skin, the larger β-irradiation field area, the higher the incidence of skin disorders. Similar results have been confirmed by other experimental systems^8,9^. This radiation-induced field size effect (RIFSE) indicates that radiation-induced biological damage is not simply proportional to the dose in the cell tissue, but depends on the irradiation field size and volume, which provides information for radiological protection problems of localized exposure.

In 2019, Matsuya et al. investigated the dependence of the induction of DSB (DNA double strand break) on the cumulative absorbed dose in normal human lung cells under localized chronic exposure with Cs-bearing particles composed of ^137^Cs and ^134^Cs attached to the cell surface compared with uniform irradiation with ^137^Cs γ-rays^5^. They reported that the number of DSB increased with uniform irradiation, but for the local irradiation, the DSB was nearly constant regardless of the dose. In addition, to demonstrate the effects of non-uniform exposure, a half-field (with 50% in the culture dish irradiated with 1 Gy of X-ray) experiment was conducted, and they observed that the amount of DSB was reduced compared to uniform irradiation.

In recent years, evidence has accumulated for a radiation-induced rescue effect (RIRE), which refers to a phenomenon in which detrimental effects in targeted irradiated cells are reduced upon receiving feedback signals from partnered non-irradiated bystander cells, or from medium previously conditioned by these bystander cells^10–14^. Thus, local irradiation produces a mixture of irradiated cells and non-irradiated cells, so irradiated cells may be rescued by the existence of non-irradiated cells near the irradiated cells.

Although these reports are very important in considering the health risks of internal exposure from the perspective of radiation protection, the biological mechanism has not been clarified.

Recently, the application of microbeam irradiation techniques in biology has attracted much interest, such techniques make it possible to irradiate individual cells with X-rays with precise dose control^15^. In particular, X-ray microbeam system using synchrotron radiation having the advantage of its small divergence, the beam size, that is, the irradiated area can be changed by cutting out the X-ray beam^16–20^. Thus, X-ray microbeam irradiation is very effective to reproduce the heterogeneous dose distribution of cell population following internal exposure. In this study, we investigated whether the DNA damage and cell proliferation response of normal human cells differ depending on the X-irradiated field size of a cell population.

## Materials and methods

### Cell culture

Primary normal human fibroblasts from the lung, MRC-5, were obtained from the European Collection of Authenticated Cell Cultures (ECACC, UK). MRC-5 cells were cultured in Basal Medium Eagle (BME) (SH30157.01, HyClone) supplemented with 10% fetal bovine serum (FBS) (S1820-500, Biowest) and penicillin–streptomycin at 37 °C in a humidified incubator with 5% CO_2_.

### Preparation of cell population

**Figure 1:**
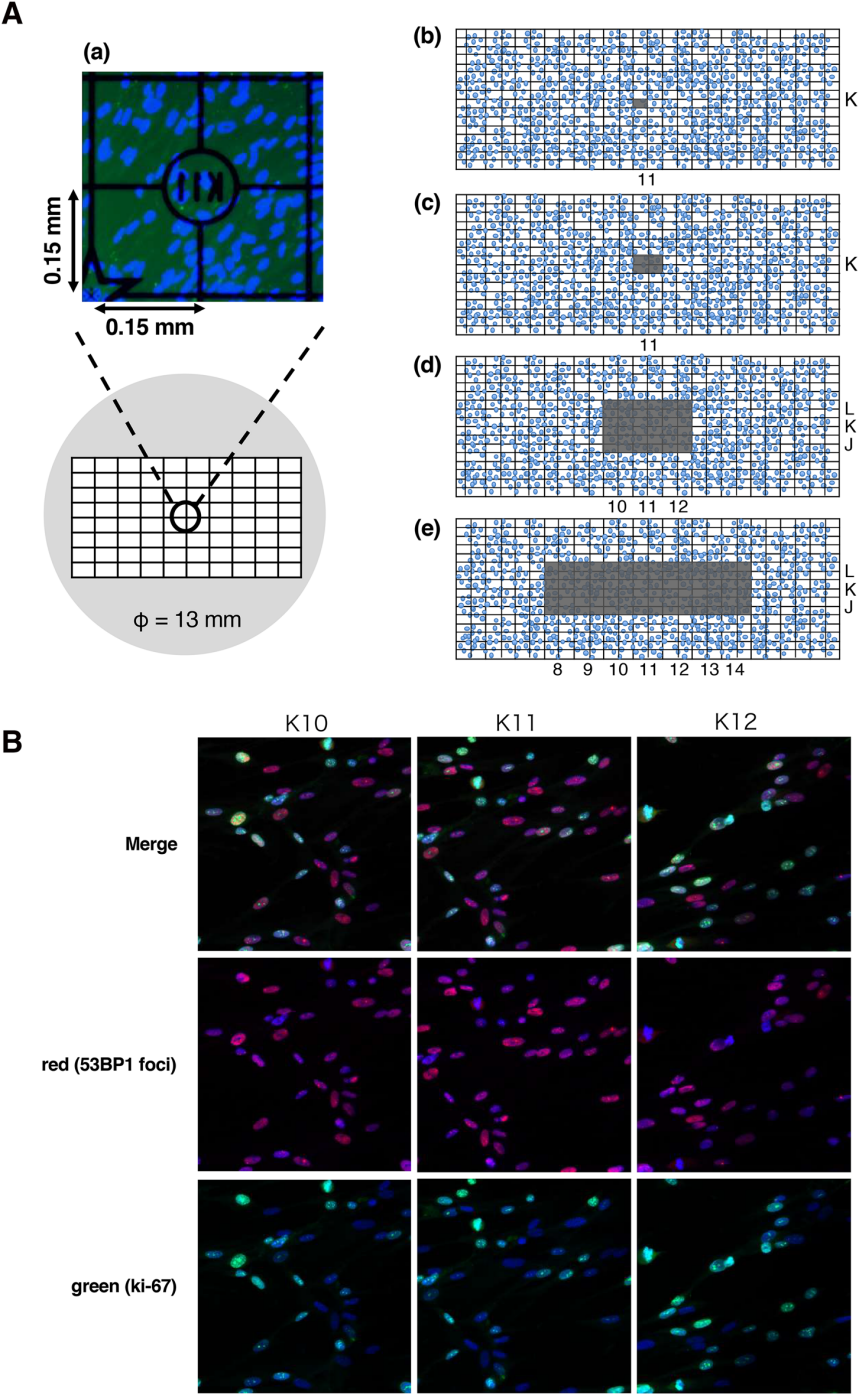
Schematic representation of X-ray microbeams for different field sizes and representative immunofluorescence images of 53BP1 foci and Ki-67-positive cell. (**A**) The illustration of a cover glass with grid lines and X-irradiation field sizes. The cells were cultured on a cover glass with grid lines to create the cell populations. This cover glass is a 0.15-mm grid that is engraved on the surface of the cover glass that makes it possible to confirm the irradiation field in more detail (a). X-ray microbeams were applied at 0.02 mm^2^ (b), 0.09 mm^2^ (c), 0.81 mm^2^ (d), and 1.89 mm^2^ (e) irradiation field sizes to cell populations by an X-ray microbeam generator, centering on K11 on the cover glass. Irradiation field is shown in gray. (**B**) The representative immunofluorescence image of the X-irradiated area (K11) and the non-irradiated area (K10, K12) in 0.09 mm^2^. Blue: Nuclei. Red: 53BP1 foci. Green: Ki-67-positive cells. These figures were created with PowerPoint for Mac ver.16.46.

In this study, we used a cover glass with a grid line (GC1300, Matsunami Glass Industry Co., Ltd.). The 0.15-mm grid engraved on the surface of the cover glass makes it possible to confirm the position of the X-irradiated cells under a microscope in detail (Fig. 1A(a)).

MRC-5 cells were cultured on the cover glass in BME supplemented with 10% FBS and penicillin–streptomycin at 37 °C in a humidified incubator with 5% CO_2_ to prepare a cell population of 133 mm^2^.

### Defined size X-ray microbeam irradiation

Defined size monochromatic X-rays at 5.35 keV were delivered by synchrotron X-ray microbeam irradiation system installed at the BL-27B station in the Photon Factory, KEK^18–21^. The beam size can be arbitrarily changed with a precision slit installed in the system. The cell population on the cover glass, placed on a 35-mm plastic dish, was irradiated by X-ray with different beam area from 0.02- to 1.89-mm^2^ (Fig. 1A(b-e)). Prior to irradiation, the beam image was acquired by a scintillator crystal, CaF_2_(Eu) (Ohyo Koken Kogyo Co., Ltd.), placed at the sample stage, and the center of the beam image was recorded. The 35-mm dish with the cover glass was turned upside down after removing the medium, placed on the sample stage. The motorized sample stage was moved to align the sample center with the recorded beam center and irradiated with X-rays from below. Due to the small amount of remaining medium between the cover glass and 35-mm dish, the cover glass stuck to the plastic surface and did not move or fall off during irradiation.

The X-ray dose at the sample position was measured with a photodiode (IRD AXUV-100G). Typically, the photon fluence was 2 × 10^8^ photons/1.89-mm^2^/s or 10^14^ photons/m^2^/s. The uniformity of the beam is confirmed by the intensity profile in the beam image obtained by the scintillator. This fluence is equivalent to the dose of 0.25 Gy/sec of 5.35 keV X-rays and the dose received by each cell does not change when the beam size is changed.

The 5-keV X-rays interact with biological samples including water, almost exclusively through the photoelectric effect. Because of the range of secondary photoelectrons with an energy of 5-keV is known to be 0.8 μm, the energy deposited outside the beam area is considered to be very small.

### Immunofluorescence assay

#### 53BP1

DSB is one of the initial DNA damages of radiation. When DSB occur in cell nucleus, one of the DSB repair proteins, 53BP1 (p53 Binding Protein 1), accumulates at the cleavage site and forms foci. This focus can be visualized by immunofluorescence staining and can be used as an indicator of DSB^22^. Therefore, in this study, immunofluorescence staining of 53BP1 was performed to detect DSB. In brief, the cells were fixed with 100% methanol for 15 min on ice. After fixation, the cells were rinsed with phosphate buffered saline (PBS) and permeabilized in 0.5% TritonX-100 in PBS for 5 min on ice, and then washed extensively with PBS. The cells were incubated with an anti-53BP1 polyclonal antibody (A300-272A, Bethyl Laboratories) diluted 1:1000 by blocking solution (3% bovine serum albumin (BSA) in PBS) for 24 h at 4 °C as a primary antibody, then washed with PBS twice. Thereafter, the cells were incubated with Alexa Fluor 546-conjugated donkey anti-rabbit IgG (A10040, Invitrogen) diluted 1:200 by blocking solution for 1 h at room temperature (RT) as a secondary antibody. After washing with PBS three times, 10 μL of DAPI (H-1200, VECTOR laboratories) was dropped on a slide glass, and the cover glass with cells was attached to the slide glass. 53BP1 foci were observed under a fluorescence microscope (IX81; Olympus, Japan) as shown by red signal in Fig. 1B, and the number of 53BP1 foci per cell was counted per irradiated area.

#### Ki-67

Ki-67 is a nuclear protein that is expressed during all active phases of the cell cycle (G_1_, S, G_2_, and M), but is not expressed in resting cells (G_0_). Thus, Ki-67 can be used as a marker for cell proliferation. In brief, cells were permeabilized with 0.1% TritonX-100 in PBS on ice for 10 min, then blocked with 2% BSA for 10 min at RT. The cells were labeled with a Ki-67 monoclonal antibody (14-5698-82, eBioscience) diluted 1:200 by blocking solution, incubated at 4 °C overnight, and then labeled with Alexa Fluor 488-conjugated goat anti-rat antibody (A-11006, Invitrogen) diluted 1:200 by blocking solution for 1 h at RT as a secondary antibody. Nuclei were stained with DAPI (H-1200, VECTOR laboratories). The signal of Ki-67 was observed under a fluorescence microscope (IX81; Olympus, Japan) as shown by green signal in Fig. 1B, and the frequency of Ki-67-positive cells was counted per irradiated area.

### Cellular DNA damage depending on the field size of the X-irradiated cell population

To analyze the difference in cellular DNA damage depending on the X-irradiated field size of the cell population, field sizes of 0.02 mm^2^, 0.09 mm^2^, 0.81 mm^2^, and 1.89 mm^2^ on the cell population were irradiated with X-rays of 1 Gy. The numbers of DSB per cell were measured by immunofluorescence staining of 53BP1 for up to 48 h post-irradiation.

### Cell proliferation depending on the field size of the X-irradiated cell population

To analyze the difference in cell proliferation depending on the X-irradiated field size of the cell population, the same field sizes and X-ray dose as in the above DSB measurement were used. The frequency of proliferating cells was measured by immunofluorescence staining of Ki-67 at both 24 h and 48 h after X-irradiation.

### Statistical methods

Generalized linear models were used to statistically analyze the counts (y) of DSB among observed cells (z). Poisson regression analysis was conducted by R packages (version 4.0.2)^23^. The effect of X-irradiated field size of the cell population, 0.02 mm^2^, 0.09 mm^2^, 0.81 mm^2^ and 1.89 mm^2^, was investigated using the model that was expressed by a log-linear function with quadratic terms of the field sizes (m);

$$log\left(\frac{y}{z}\right)=a\bullet {m}^{2}+b\bullet m+c$$

where y and z are the number of 53BP1 foci and observed cells, respectively. In order to examine the statistical difference between field sizes, an additional variable was also modeled. The log-likelihood ratio test or AIC was used to provide evidence against the reduced model in favor of a model.

## Results

### Cellular DNA damage depending on the X-irradiated field size

**Figure 2:**
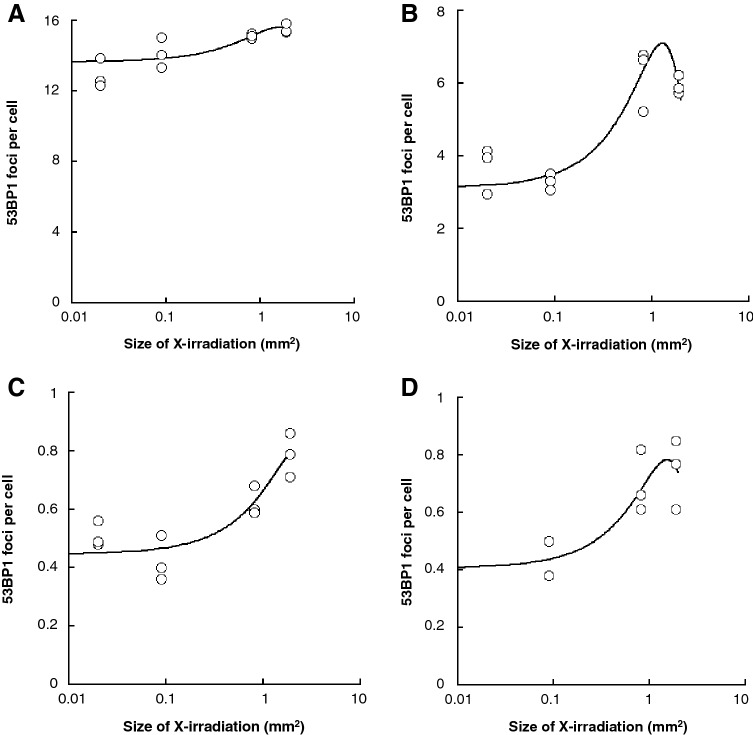
Effects of X-irradiated field sizes on the DNA damage response. The cells were irradiated with 1 Gy of X-rays at a dose rate of 0.25 Gy/s. The number of 53BP1 foci as DSB per cell was measured at 1 h (**A**), 4 h (**B**), 24 (**C**) and 48 h (**D**) post-irradiation. The data were obtained from three or four independent experiments and the mean values for each experiment were plotted. Open circles indicate the number of 53BP1 foci per cell in X-irradiated cells. The change in the number of 53BP1 foci depending on the field size were plotted with the best estimates (solid line) using the generalized linear models. These figures were created with KaleidaGraph ver.4.5.2.

Figure 2 shows the number of DSB per cell in X-irradiated cell populations at 1–48 h after X-irradiation of 1 Gy on field sizes of 0.02–1.89 mm^2^. The mean number of DSB in sham-irradiated controls was 0.56 ± 0.09 per cell and was almost constant for 48 h. The mean number of DSB per cell was 12.92 ± 0.82 in 0.02 mm^2^, 14.14 ± 0.85 in 0.09 mm^2^, 15.12 ± 0.14 in 0.81 mm^2^, and 15.51 ± 0.29 in 1.89 mm^2^ at 1 h after X-irradiation (Fig. 2A). After incubation to allow DNA damage repair, the mean number of DSB per cell was 3.68 ± 0.64 in 0.02 mm^2^, 3.30 ± 0.19 in 0.09 mm^2^, 6.21 ± 0.86 in 0.81 mm^2^, and 5.94 ± 0.25 in 1.89 mm^2^ at 4 h (Fig. 2B); 0.51 ± 0.04 in 0.02 mm^2^, 0.44 ± 0.08 in 0.09 mm^2^, 0.62 ± 0.05 in 0.81 mm^2^, and 0.79 ± 0.08 in 1.89 mm^2^ at 24 h (Fig. 2C); and 0.44 ± 0.09 in 0.09 mm^2^, 0.70 ± 0.11 in 0.81 mm^2^, and 0.74 ± 0.12 in 1.89 mm^2^ at 48 h (Fig. 2D). At each time point, the best fitted model based on AIC was (y/z) = exp{a * (field size) ^2^ + b*(field size) + c}, where y and z are the number of 53BP1 foci and observed cells, respectively. The estimates of the parameters are: a = − 0.0518 (95%CI − 0.082, − 0.021), b = 0.167 (95%CI 0.093, 0.241) and c = 2.61(95%CI 2.58, 2.65) at 1 h after X-irradiation; a = − 0.495 (95%CI − 0.534, − 0.457), b = 1.28 (95%CI 1.18, 1.37), and c = 1.14 (95%CI 1.08, 1.18) at 4 h; a = − 0.103 (95%CI − 0.209, 0.002), b = 0.498 (95%CI 0.250, 0.752), and c = − 0.808 (95%CI − 0.931, − 0.688) at 24 h; a = − 0.278 (95%CI − 0.387, − 0.173), b = 0.855 (95%CI 0.585, 1.14), and c = − 0.901 (95%CI − 1.052, − 0.756) at 48 h.

The likelihood ratio test provided a statistically significant improvement by the model with the linear and quadratic terms of field size (p < 0.01). At 24 h, there was marginal significance (p = 0.053), while AIC (133.72) of the model with quadratic term was smaller than AIC (135.45) of the linear model. At each time point, the overall trend of the frequency of DSB against field size showed a strong dependence on field size. The frequency appeared to converge to the value at a large field size since the negative value of the quadratic term was estimated.

**Figure 3:**
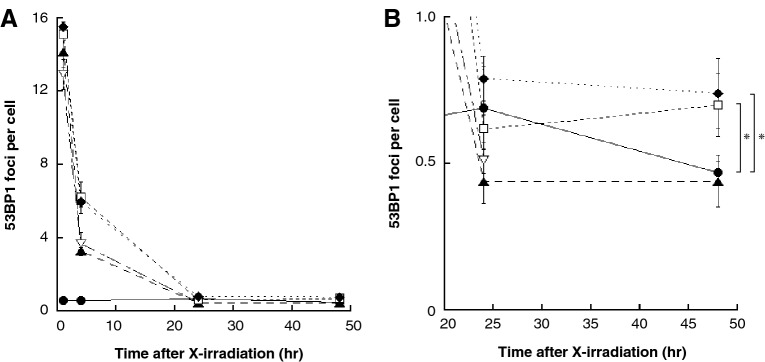
DSB repair curves for each X-irradiated field size. (**A**) The DSB repair curves were created by combining A-D in Fig. 2. The results were represented as mean ± standard error. Lower triangle, Triangle, Square, and Rhombus indicates the number 53BP1 foci per cell of X-irradiated cells in 0.02 mm^2^, 0.09 mm^2^, 0.81 mm^2^, and 1.89 mm^2^, respectively. The number of 53BP1 foci per cell in sham-irradiated controls is Closed circle. (**B**) The DSB repair curves focused on 24- to 48-h after X-irradiation. At 1.89 mm^2^ and 0.81 mm^2^, the number of 53BP1 foci were significantly higher than background level at 48 h after X-irradiation (p < 0.05, t-test). These figures were created with KaleidaGraph ver.4.5.2.

The time-response curve of DNA damage is shown in the Fig. 3. With field sizes of 0.09 or under, the mean number of DSB per cell decreased rapidly and fell below the background level at 24 h after X-irradiation (Fig. 3A). However, with field sizes of 0.81 or over, the mean number of DSB per cell remained even 48 h after X-irradiation (p < 0.05, t-test) (Fig. 3B).

**Figure 4:**
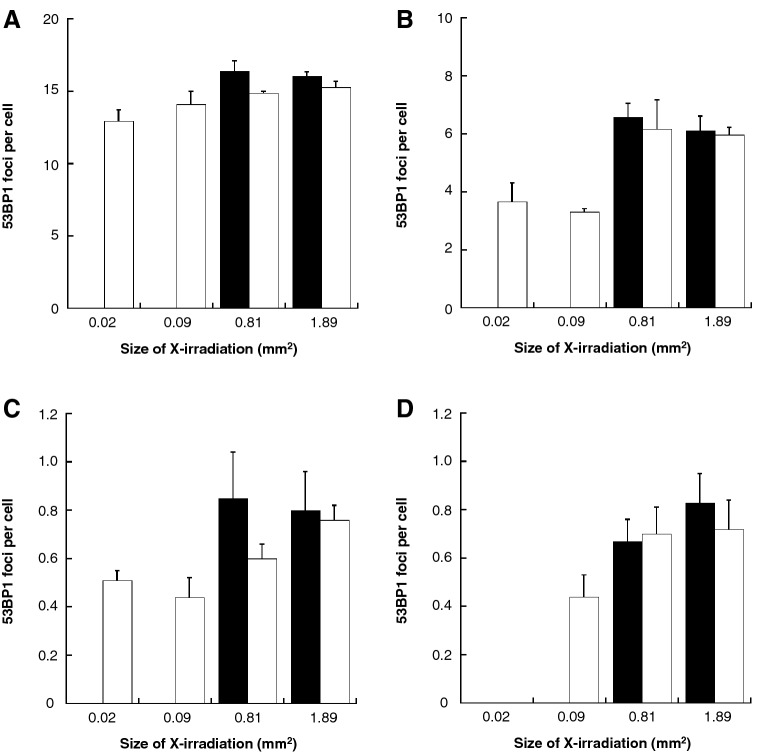
X-irradiated field area distribution of cells with DNA damage in the cell population. The X-irradiated field sizes of 0.81 mm^2^ and 1.89 mm^2^ were divided into 0.3 mm × 0.3 mm, and the X-irradiated field area distribution of cell with DNA damages in cell population was analyzed at 1 h (**A**), 4 h (**B**), 24 h (**C**), 48 h (**D**), post-irradiation. After that, we investigated the number of 53BP1 foci as DSB per cell in cells located in area having not contact (black bar) or contact (white bar) with non-irradiated area of a cell population. Since the areas with X-irradiated field seizes of 0.02 mm^2^ and 0.09 mm^2^ are totally in contact with non-irradiated area, there is no data for the area in not contact with non-irradiated area. The data were pooled from three or four independent experiments and the results were represented as mean ± standard error. These figures were created with KaleidaGraph ver.4.5.2.

In addition, we analyzed the number of DSB in cells located in area having not contact or contact with non-irradiated area of a cell population X-irradiated with 0.02–1.89 mm^2^ (Fig. 4). The mean number of DSB in cells located in area having not contact with non-irradiated area were respectively (black bar in Fig. 4): 16.37 ± 0.75 in 0.81 mm^2^, 16.05 ± 0.28 in 1.89 mm^2^ at 1 h (Fig. 4A); 6.58 ± 0.49 in 0.81 mm^2^, 6.10 ± 0.51 in 1.89 mm^2^ at 4 h (Fig. 4B); 0.85 ± 0.19 in 0.81 mm^2^, 0.80 ± 0.16 in 1.89 mm^2^ at 24 h (Fig. 4C); and 0.67 ± 0.09 in 0.81 mm^2^, 0.83 ± 0.12 in 1.89 mm^2^ at 48 h (Fig. 4D). The mean number of DSB in cells located in area having contact with non-irradiated area were respectively (white bar in Fig. 4): 12.92 ± 0.82 in 0.02 mm^2^, 14.14 ± 0.85 in 0.09 mm^2^, 14.87 ± 0.12 in 0.81 mm^2^, 15.29 ± 0.38 in 1.89 mm^2^ at 1 h (Fig. 4A); 3.68 ± 0.64 in 0.02 mm^2^, 3.30 ± 0.11 in 0.09 mm^2^, 6.16 ± 1.01 in 0.81 mm^2^, 5.96 ± 0.26 in 1.89 mm^2^ at 4 h (Fig. 4B); 0.51 ± 0.04 in 0.02 mm^2^, 0.44 ± 0.08 in 0.09 mm^2^, 0.60 ± 0.06 in 0.81 mm^2^, 0.76 ± 0.06 in 1.89 mm^2^ at 24 h (Fig. 4C); and 0.44 ± 0.09 in 0.09 mm^2^, 0.70 ± 0.11 in 0.81 mm^2^, 0.72 ± 0.12 in 1.89 mm^2^ at 48 h (Fig. 4D).

The cells located in areas contact with non-irradiated area tended to have a lower number of DSBs.

### Cell proliferation depending on X-irradiated field size

**Figure 5:**
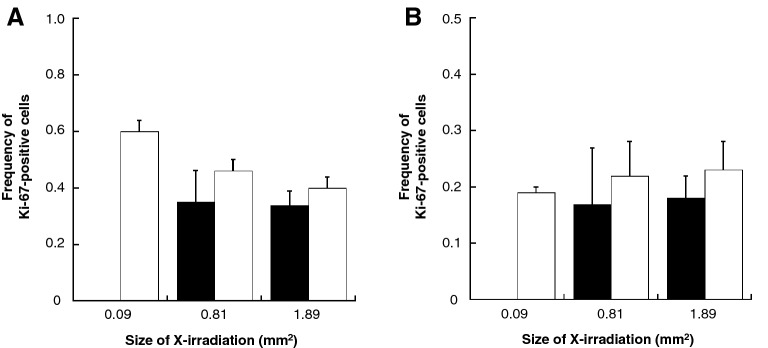
X-irradiated field area distribution of Ki-67-positive cells. X-irradiated field sizes of 0.81 mm^2^ and 1.89 mm^2^ were divided into 0.3 mm × 0.3 mm, and the X-irradiated field area distributions of Ki-67-positive cells were analyzed at 24 h (**A**) and 48 h (**B**) post-irradiation. After that, we investigated the frequency of Ki-67-positive cells located in area having not contact (black bar) or contact (white bar) with non-irradiated area of a cell population. However, since the areas with X-irradiated field seizes of 0.09 mm^2^ are totally in contact with non-irradiated area, there is no data for the area in not contact with non-irradiated area. The data were pooled from three or four independent experiments and the results were represented as mean ± standard error. These figures were created with KaleidaGraph ver.4.5.2.

We analyzed the frequency of Ki-67-positive cells located in area having not contact or contact with non-irradiated area of a cell population X-irradiated with 0.09–1.89 mm^2^. The mean frequencies of Ki-67-positive cells in cells located in area having not contact with non-irradiated area were respectively (black bar in Fig. 5): 0.35 ± 0.11 in 0.81 mm^2^, 0.34 ± 0.05 in 1.89 mm^2^ at 24 h; and 0.17 ± 0.01 in 0.81 mm^2^, 0.18 ± 0.04 in 1.89 mm^2^ at 48 h (Fig. 5A). The mean frequencies of Ki-67-positive cells in cells located in area having contact with non-irradiated area were respectively (white bar in Fig. 5): 0.60 ± 0.04 in 0.02 mm^2^, 0.46 ± 0.04 in 0.81 mm^2^, 0.40 ± 0.04 in 1.89 mm^2^ at 24 h; and 0.19 ± 0.01 in 0.09 mm^2^, 0.22 ± 0.06 in 0.81 mm^2^, 0.23 ± 0.05 in 1.89 mm^2^ at 48 h (Fig. 5B).

## Discussion

When considering the health risks of internal exposure, characterizing the response of cell populations to local exposure is of significant radiological importance. The aim of this study was to assess whether the cellular response to DNA damage depends on the X-irradiated field size of the cell population.

The first key finding from this study is that, even at the same dose, the number of DSB per cell increases depending on the X-irradiated field size on the cell population (open circle in Fig. 2). Thus, RIFSE was confirmed in this study. However, RIFSE reached a plateau level above 0.81 mm^2^ of X-irradiated field size on the cell population. As a phenomenon that supports this result, Lam et al. irradiated α-rays on 2.5–100% of cells in a population and examined the frequency of DSB at 12 h after α-irradiation^12^. As a result, they observed that the lower the proportion of α-irradiated cells, the lower the number of DSB. They also reported that the number of DSB plateaued when more than 75% of cells were irradiated with α-rays. A similar result was reported by Butterworth et al.^24^. They irradiated 25–100% of cell population with X-rays and showed that the survival ratio of X-irradiated cells tended to increase with the decrease in X-irradiated cell population, and the survival rate significantly increased at 25% compared to 100% irradiation. However, there was no difference in cell viability at above a 75% X-irradiated cell population. From these results, we infer that the response of a cell population to radiation changes depends on the ratio of irradiated cells, and that it plateaus when a certain ratio of cells is irradiated.

The second key finding of this study is that the number of DSB tended to decrease in cells at the area in contact with non-irradiated area of the X-irradiated cell population (white bar in Fig. 4). In this study, X-irradiated field sizes were divided into 0.3 mm × 0.3 mm, and the X-irradiated field area distribution of cells with DNA damage in each population was analyzed. As a result, we found that X-irradiated cells in area in contact with non-irradiated area tended to repair DNA damage smoothly, and in the 1.89 mm^2^ X-irradiation field size, DNA damage seemed to be persistent in cells located in area having not contact with non-irradiated area of the irradiated population (black bar in Fig. 4D). This result indicated that DNA damage repair in X-irradiated cells was enhanced when X-irradiated cells were surrounded by non-irradiated cells. In 2011, Chen et al. reported that when human cervical cancer cells were irradiated with 20 cGy of α-rays and co-cultured with non-irradiated human primary fibroblast cells, the number of DSB in the α-irradiated cells was less than that in α-irradiated cells that were not co-cultured with non-irradiated cells at a statistically significant level. Similarly, micronucleus formation in α-irradiated cells and the number of annexin V-positive apoptotic cells upon α-irradiation were reduced in the presence of non-irradiated cells^10^. This phenomenon is called the radiation-induced rescue effect (RIRE), in which detrimental effects in targeted irradiated cells are reduced upon receiving feedback signals from partnered non-irradiated bystander cells, or from medium previously conditioned with these partnered non-irradiated bystander cells. Since then, a number of important advances have been made in RIRE research that have highlighted the importance of radiation biology^14^. From these reports, we thought that RIRE occurs in cells at the boundary between the X-irradiated field and the non-irradiated field. Thus, we hypothesized that, in a small irradiation field size, most X-irradiated cells are in contact with non-irradiated cells, which can lead to RIRE and, so RIRE is involved in RIFSE. RIRE is also mediated by the secreted factors from bystander neighboring cells. Since the diffusion of the bystander signal mediators via a culture medium needs some time^25^, in a large irradiation field size, RIRE may be delayed in X-irradiated cells at the area in not contact with non-irradiated area. In addition, the larger irradiation field size, the more susceptible the center area of X-irradiated cell populations is to the bystander signal, which may delay DNA damage repair.

The third key finding of this study is that an increase in the frequency of Ki-67-positive cells can be observed in cells located in area having contact with non-irradiated area of an X-irradiated cell population regardless of the X-irradiated field size (white bar in Fig. 5). In recent years, microbeam radiotherapy (MRT) based on the tissue-sparing effect (TSE) in response to non-uniform radiation fields has attracted attention^26–28^. MRT can be effective at destroying tumors because a collimator subdivides the homogeneous radiation field into an array of co-planar, high-dose beams that are tens of micrometers wide and separated by a few hundred micrometers that cause very little damage to normal tissues. In 2019, Fukunaga et al. used a combination of MRT techniques and a unique ex vivo testes organ culture to perform high-precision 200, 50, and 12.5 μm-slit irradiation where approximately 50% of the sample was irradiated via a four-dimensional slit system with a monochromatic X-ray microbeam irradiator to investigate the biological responses of non-uniform radiation fields^26^. They revealed that the survival and potential migration steps of the non-irradiated germ stem cells in the irradiated testes tissue are needed for effective TSE for spermatogenesis. Extensive cell migration between the irradiated- and non-irradiated-areas after MRT has also been reported by Crosbie et al.^29^. In the present study, the field area distribution of Ki-67-positive cells in the cell populations was also analyzed, and we found that Ki-67-positive cells tended to increase in the area in contact with non-irradiated areas. Thus, our results imply that, in a small X-irradiated field size, cells in the non-irradiated area migrated to the irradiated area and contributed to the regeneration of the cell population.

The results of the present study suggest that RIFSE is also observed in normal human fibroblast cells by X-irradiation. Furthermore, we speculate that RIRE might be involved in the response of cell populations post-irradiation, and is thus very important when considering the health risks of internal exposure and MRT.

## Conclusions

We identified the RIFSE where the number of DSB increases with the X-irradiated field size of the cell population. In addition, the number of DSB tended to decrease in cells located in area having contact with non-irradiated area. This result indicated that DNA damage repair in X-irradiated cells was enhanced when X-irradiated cells were surrounded by non-irradiated cells. This study suggests that X-irradiated cells received some signal (a rescue signal) from surrounding non-irradiated cells may be involved in the response of cell populations post-irradiation.
